# Combating Foodborne MRSA: Identification and Silver Nanoparticle-Based Antibacterial Strategies with Antibiotic Synergy and Resistance Evolution Assessment

**DOI:** 10.3390/microorganisms13102393

**Published:** 2025-10-18

**Authors:** Adil Abalkhail, Eman Marzouk

**Affiliations:** Department of Public Health, College of Applied Medical Sciences, Qassim University, P.O. Box 6666, Buraydah 51452, Saudi Arabia

**Keywords:** *Staphylococcus aureus*, methicillin resistant *Staphylococcus aureus*, silver nanoparticles, antibiotic synergy, ready-to-eat foods, public health

## Abstract

Ready-to-eat (RTE) foods can carry antimicrobial-resistant pathogens; however, few studies link real-world surveillance to practical interventions. This study addressed this gap by estimating the prevalence of *Staphylococcus aureus* (*S. aureus*) and methicillin-resistant *S. aureus* (MRSA) in ready-to-eat foods from Al-Qassim and evaluating a rapid, orthogonal confirmation workflow (culture → MALDI-TOF MS → Vitek 2 → *mecA*/*mecC* PCR). The in vitro activity of citrate-stabilized silver nanoparticles (AgNPs) against food-derived MRSA was quantified, and synergy with oxacillin (primary) and ciprofloxacin (secondary) was examined. Silver-susceptibility stability was assessed over 20 days of sub-MIC serial passage, with attention to whether β-lactam co-exposure constrained drift. We surveyed 149 RTE products and paired the confirmation workflow with mechanistic tests of AgNPs as antibiotic adjuvants. *S. aureus* was recovered from 24.2% of products and MRSA from 6.7%, with higher recovery from animal-source matrices and street-vendor outlets. MALDI-TOF MS provided rapid species confirmation and revealed two reproducible low-mass peaks (*m*/*z* 3990 and 4125) associated with MRSA, supporting spectral triage pending molecular confirmation. Antimicrobial susceptibility testing showed the expected β-lactam split (MRSA oxacillin/cefoxitin non-susceptible; MSSA oxacillin-susceptible but largely penicillin-resistant), with last-line agents retained. Citrate-stabilized AgNPs displayed consistent potency against food-derived MRSA (MIC 8–32 µg/mL; MIC_50_ 16; MIC_90_ 32) and were predominantly bactericidal (MBC/MIC ≤ 4 in 90%). Checkerboards demonstrated frequent AgNP–oxacillin synergy (median fractional inhibitory concentration index [FICI] 0.37; 4–16-fold oxacillin MIC reductions) and additive-to-synergistic effects with ciprofloxacin (median FICI 0.63), translating time–kill assays into rapid, sustained bactericidal activity without antagonism. During sub-MIC evolution, silver MICs rose modestly (median two-fold) and often regressed off drug; oxacillin co-exposure limited drift. RTE foods therefore represent credible MRSA exposure routes. Integrating MALDI-assisted triage with automated AST enables scalable surveillance, and standardized AgNP formulations emerge as promising β-lactam adjuvants—pending in situ efficacy, safety, and residue evaluation.

## 1. Introduction

Methicillin-resistant *Staphylococcus aureus* (MRSA) remains a high-priority pathogen at the human–food–environment interface [1,2]. Beyond its clinical burden, converging evidence shows that *Staphylococcus aureus* (*S. aureus*) including MRSA contaminates diverse ready-to-eat (RTE) foods, positioning food as a plausible vehicle for exposure and dissemination across One Health sectors [3,4]. Systematic reviews and meta-analyses report substantial *S. aureus* contamination in RTE categories and meats, with non-trivial pooled MRSA prevalence in animal-derived foods [5]. Complementary multi-country and regional assessments likewise document global MRSA contamination in meat products, broader *S. aureus* occurrence across food matrices including RTE items, and European surveillance confirming MRSA detection within harmonized monitoring frameworks [6,7,8].

Risk interpretation is nuanced. Authoritative reviews note that direct evidence for human infection via consumption of MRSA-contaminated foods remains limited; nonetheless, food can serve as a contamination source and reservoir for livestock-associated lineages. Genomic and epidemiologic investigations document transmission at animal–human interfaces (occupational and household) and suggest occasional foodborne routes [9,10].

Global prioritization initiatives consistently rank *S. aureus*—including resistant forms—among major public health concerns, reflecting high burden, transmission potential, and constrained therapeutic options. Large-scale burden analyses place *S. aureus*, especially MRSA, among leading contributors to deaths attributable to antimicrobial resistance (AMR), with regional assessments corroborating substantial MRSA-attributable deaths and disability-adjusted life years (DALYs) [11,12,13].

Robust identification of foodborne MRSA benefits from complementary platforms that combine speed, specificity, and confirmatory power. Matrix-assisted laser desorption/ionization time-of-flight (MALDI-TOF) mass spectrometry enables rapid species-level identification and, in exploratory workflows, has reported spectral patterns associated with MRSA versus methicillin-susceptible *S. aureus* (MSSA); however, performance varies by protocol and dataset, and reliable resistance determination still requires molecular confirmation [14,15]. Automated antimicrobial susceptibility testing (AST) systems such as Vitek 2 provide high categorical/essential agreement for *Staphylococcus* susceptibility testing and can support early MRSA flagging at scale [16,17].

Definitive confirmation relies on PCR targeting *mecA*/*mecC* (and, where appropriate, SCCmec–orfX junction assays) and may be complemented by penicillin-binding protein 2a (PBP2a) antigen detection in selected workflows; these remain contemporary reference standards in MRSA diagnostics [18]. Best practice screening uses cefoxitin as a surrogate for PBP2a-mediated resistance with harmonized CLSI/EUCAST breakpoints and quality control; nevertheless, *mecC*-associated detection gaps and occasional phenotype–genotype discrepancies warrant confirmatory *mecA*/*mecC* testing when discordance or epidemiologic significance is suspected [19,20].

Against the backdrop of escalating AMR, silver nanoparticles (AgNPs) exhibit a multitarget antibacterial mode of action in *S. aureus*—including membrane disruption, metabolic/proteostasis collapse, nucleic-acid interactions, and reactive-oxygen-species effects—and can potentiate conventional antibiotics. Checkerboard and time–kill studies report AgNP–antibiotic synergy with biofilm suppression, particularly with β-lactams, supporting their evaluation as adjuvants to restore or enhance drug efficacy [21,22,23]. Synergistic strategies that pair AgNPs with antibiotics—and, in some cases, adjunct modalities such as visible blue light or plant-derived polyphenols—produce augmented bactericidal activity against MRSA and biofilm-forming *S. aureus*, reinforcing the translational rationale for AgNP-enabled combination therapy [21,24,25].

Durability of AgNP efficacy, however, cannot be assumed. Experimental evolution and serial passage studies show that *S. aureus* can increase minimum inhibitory concentrations (MICs) under repeated sub-inhibitory exposure—potentially via nanoparticle aggregation driven by enhanced biofilm formation and secreted factors—while covalent immobilization of silver on cyanographene has restored activity against AgNP-resistant phenotypes [26,27]. Conversely, other work reported no stable resistance after 16 serial passages with Ag^+^ or AgNPs, highlighting context-dependent outcomes and the need for long-term tests in food-derived MRSA under clinically relevant AgNP ± antibiotic regimens [21]. To date, few studies have simultaneously tracked long-term AgNP susceptibility in food-derived MRSA while quantifying AgNP–antibiotic interactions under standardized conditions.

From a One Health perspective, heavy metals and other non-antibiotic stressors may co-select for antibiotic resistance, shaping antimicrobial-resistance-gene persistence in food-processing environments and along supply chains. Understanding how silver-based interventions intersect with such co-selection dynamics is essential for risk-aware deployment in food safety and clinical contexts [28].

Accordingly, the present study estimated the prevalence of *S. aureus*/MRSA in RTE foods from Al-Qassim; evaluated a rapid, orthogonal confirmation workflow (culture → MALDI-TOF MS → Vitek 2 → *mecA*/*mecC* PCR); quantified the in vitro activity of citrate-stabilized AgNPs and their interactions with oxacillin and ciprofloxacin against food-derived MRSA; and assessed the stability of silver susceptibility during 20 days of sub-MIC serial passage, including whether β-lactam co-exposure constrains drift. By integrating surveillance with standardized confirmation, synergy testing, and sub-MIC evolution assays, this work provides translational evidence at the food–human interface to inform One Health-aligned AMR-mitigation strategies in the RTE sector.

## 2. Materials and Methods

### 2.1. Study Design and Sampling

This cross-sectional survey was carried out in the Al-Qassim region of Saudi Arabia from December 2024 to April 2025. A total of 149 RTE items were collected using a cross-stratified approach by food category—meat-based (n = 40), poultry-based (n = 32), salads/vegetables (n = 28), dairy-based (n = 25), and bakery/sweets (n = 24)—and by point of sale—restaurants/takeaways (n = 60), supermarkets/delis (n = 54), and street vendors/markets (n = 35) (Table 1). Purchases were distributed across multiple municipalities and staggered across different days and districts to reduce clustering and improve representativeness. All items were transported in insulated coolers maintained at ≤ 8°C and were processed within 4 h of purchase.

### 2.2. Culture and Preliminary MRSA Screening

Food homogenates were pre-enriched in Tryptic Soy Broth (TSB; Oxoid, Thermo Fisher Scientific, Altrincham, UK) supplemented with 6.5% NaCl (Sigma-Aldrich/Merck, Darmstadt, Germany) at a 1:10 ratio (25 g in 225 mL) and incubated at 37 °C for 18–24 h. Enrichments were streaked onto Baird–Parker agar with egg yolk tellurite emulsion (Oxoid, Thermo Fisher Scientific, UK) and CHROMagar™ MRSA (CHROMagar, Paris, France), followed by incubation at 37 °C for 24–48 h. Up to three colonies per sample with morphology compatible with *Staphylococcus aureus* were purified on Tryptic Soy Agar (TSA; Oxoid, Thermo Fisher Scientific, UK) and evaluated by Gram stain (Remel Gram Stain Kit, Thermo Fisher Scientific), catalase (3% H_2_O_2_; Sigma-Aldrich/Merck), and coagulase (Oxoid Staphytect™ Plus latex agglutination, Thermo Fisher Scientific). DNase agar (Oxoid, Thermo Fisher Scientific, UK) was used when required by SOPs. Isolates meeting these criteria were classified as presumptive *S. aureus* and advanced to methicillin-resistance screening.

Preliminary MRSA screening used a cefoxitin-surrogate approach aligned with international standards. From overnight culture, a 0.5 McFarland suspension (0.85% sterile saline; Oxoid, Thermo Fisher Scientific, UK) was lawn-inoculated on Mueller–Hinton agar (MHA; Oxoid, Thermo Fisher Scientific, UK), a 30 µg cefoxitin disk (Oxoid antimicrobial susceptibility disk, Thermo Fisher Scientific) was applied, and plates were incubated at 35 ± 2 °C for 18–24 h. Zone diameters were interpreted according to contemporaneous CLSI M100 performance standards and CLSI M07 guidance, together with EUCAST breakpoints (cefoxitin as a surrogate for PBP2a-mediated resistance) [29,30]. Isolates with borderline zones, mixed phenotypes, or epidemiologically important profiles were retested and flagged for orthogonal confirmation with PBP2a antigen testing (Oxoid™ PBP2′ Latex Agglutination Test, DR0900A, Thermo Fisher Scientific) and/or *mecA*/*mecC* PCR (see Molecular Methods).

Quality control was included in every run using *S. aureus* ATCC 25923 (disk-diffusion QC), ATCC 29213 (MIC reference QC), and ATCC 43300 (MRSA, *mecA*-positive) (all from ATCC, Manassas, VA, USA). Media and disks were used within shelf life, stored per manufacturer specifications, and inspected for plate integrity before use.

### 2.3. Species Identification by MALDI-TOF MS

Species identification was performed by MALDI-TOF MS (MALDI Biotyper, Bruker Daltonics, Bremen, Germany) using MBT Compass software (v1.3) and the IVD reference library. Targets were calibrated before each run with the Bruker Bacterial Test Standard (BTS; *Escherichia coli*) according to the manufacturer’s acceptance criteria. For routine preparation, a well-isolated colony was spotted onto a polished steel target (96-well format), overlaid with 1 µL of 70% formic acid, air-dried, and overlaid with 1 µL of HCCA matrix (α-cyano-4-hydroxycinnamic acid; Bruker). Spectra were acquired in linear positive mode over 2–20 kDa using manufacturer-recommended parameters. Isolates yielding weak or ambiguous spectra were reprocessed by the ethanol–formic acid extraction protocol and reanalyzed.

The MALDI workflow served primarily to confirm *S. aureus* at the species level and secondarily, on an exploratory basis, to flag presumptive MRSA versus MSSA using a laboratory-validated rule set based on low-mass peak-intensity signatures across ≥2 independent spots. Indeterminate spectra were not called. Methicillin resistance was not assigned by MALDI; all isolates underwent cefoxitin-based phenotypic screening interpreted per contemporaneous CLSI M100/M07 and EUCAST criteria, and any resistant, discordant, or MALDI-flagged isolates were resolved by PBP2a antigen testing and/or *mecA*/*mecC* PCR (details in Molecular Methods).

### 2.4. Antimicrobial Susceptibility Testing (AST)

Phenotypic AST was performed on the VITEK^®^ 2 Compact (bioMérieux, Marcy-l’Étoile, France) using the *Staphylococcus* card AST-GP71. The panel included cefoxitin (screen), oxacillin, penicillin, erythromycin, clindamycin, gentamicin, ciprofloxacin, levofloxacin, tetracycline, trimethoprim–sulfamethoxazole, linezolid, and vancomycin. Inocula were prepared from 18 to 24 h cultures, adjusted to a 0.5 McFarland standard in sterile saline, and loaded according to the manufacturer’s instructions. Results were interpreted against contemporaneous CLSI M100 and EUCAST breakpoints.

Quality control accompanied each new lot and whenever control criteria indicated repeat testing, using *S. aureus* ATCC 29213 and ATCC 43300. For key analytes—oxacillin/cefoxitin and ciprofloxacin—reference broth microdilution (BMD; CLSI M07/M100) was performed for all isolates, and vancomycin MICs were likewise confirmed by BMD (see Section 2.5).

### 2.5. Broth Microdilution MIC and MBC Testing

Reference MICs for antibiotics and silver nanoparticles (AgNPs) were determined by BMD in cation-adjusted Mueller–Hinton broth (CAMHB; Oxoid, Thermo Fisher Scientific, UK) using sterile, low-binding 96-well plates (final well volume 100 µL). Twofold serial dilutions were prepared at 2× strength in 50 µL and mixed 1:1 with 50 µL of inoculum to yield a final concentration of 5 × 10^5^ CFU/mL. Plates were incubated at 35 ± 2 °C for 18–24 h and read visually. The MIC was defined as the lowest concentration with no visible growth. For minimum bactericidal concentration (MBC), 10 µL from non-turbid wells at or above the MIC were plated onto TSA and incubated for 24 h; the MBC was the lowest concentration producing ≥99.9% of kills relative to the starting inoculum. Unless otherwise noted, categorical and essential agreement statements refer to BMD MICs.

Antibiotic test ranges (twofold series; µg/mL): oxacillin (CAMHB + 2% NaCl; read at 24 h) 0.25–8; cefoxitin 0.5–32; vancomycin 0.125–16; linezolid 0.125–8; ciprofloxacin 0.004–2; gentamicin 0.125–32; erythromycin 0.125–64; clindamycin 0.03–16; tetracycline 0.125–64; and trimethoprim–sulfamethoxazole (1:19; reported as trimethoprim) 0.06/1.2–4/76. AgNPs: 0.5–128 µg/mL total silver (twofold series), prepared from quantified stocks and gently resuspended immediately before dispensing.

AgNP-specific precautions: CAMHB was prepared with low-chloride water; plates were protected from light; and edge wells were avoided to limit evaporation and nanoparticle ring effects. Where a solvent/dispersant was required for antibiotic stocks, the final concentration did not exceed 1% *v*/*v* and was matched in control wells. Controls included medium sterility and organism growth controls for each isolate. Visualization (checkerboard assays): Heatmaps are displayed as percent growth at 24 h with numeric color bar ticks (0, 25, 50, 75, 100%) and single-agent MICs are annotated on panel margins for reference.

### 2.6. Molecular Confirmation of MRSA

Genomic DNA was prepared by boiling lysis; a commercial kit was used for isolates with low yields. A single-tube multiplex PCR targeted *mecA*, *mecC* (also referred to as *mecAL-GA251*), *spa*, and—optionally—*lukF-PV* (PVL), using a validated scheme that distinguishes *mecA* from *mecC* while enabling simultaneous detection of *spa* and PVL [31]. Primer sets and expected amplicon sizes followed the EURL-AR MRSA Multiplex PCR-2 protocol [32]: *mecA* P4/P7 (162 bp), *mecC* MultiFP/MultiRP (138 bp), *spa* 1113F/1514R (~180–600 bp, variable), and *lukF-PV* pvl-F/pvl-R (~85 bp). The *spa* primers (1113F/1514R) derive from the standard *spa*-typing approach, and the PVL primers correspond to the original PVL detection method in *S. aureus* [33].

Thermocycling conditions were: 95 °C for 5 min; 35 cycles of 95 °C for 30 s, 55 °C for 30 s, and 72 °C for 45 s; followed by 72 °C for 5 min. Amplicons were resolved on 2% agarose and visualized with ethidium bromide (or a nonhazardous equivalent). Each run included *S. aureus* ATCC 43300 (*mecA*-positive), a confirmed *mecC*-positive external control, and a no-template control. Isolates were designated MRSA when *mecA* or *mecC* was detected at the expected size and the phenotype supported methicillin resistance. Any discordant result prompted repeat extraction and PCR, and—when indicated—PBP2a antigen testing as an orthogonal confirmatory method.

### 2.7. AgNPs: Source, Preparation, and Characterization

Citrate-stabilized, spherical AgNPs with a nominal core diameter of 10 nm (Sigma-Aldrich/Merck, cat. 730785) were used throughout. Stock dispersions (1–2 mg/mL) were stored at 4 °C, protected from light, gently resuspended immediately before use, and discarded after 30 days. Each lot was characterized before biological testing. Ultraviolet–visible (UV–Vis) spectroscopy verified the expected surface plasmon resonance for small spherical AgNPs (typically ~400–430 nm). Dynamic light scattering (DLS) provided hydrodynamic diameter and polydispersity index (PDI); zeta potential (via electrokinetic mobility) was measured to assess colloidal stability. For a subset, transmission electron microscopy (TEM) confirmed core size and morphology. Total silver in working stocks was quantified by inductively coupled plasma mass spectrometry (ICP-MS).

To minimize nanoparticle loss and artifact formation, all dilutions and measurements used low-protein-binding labware with handling aligned to dispersion-stability best practices. Methods followed standards from the International Organization for Standardization (ISO)—ISO 22412 (DLS) [34], ISO 13099-1 (zeta potential) [35], ISO 17294-2 (ICP-MS) [36]—and stability considerations were guided by the Organization for Economic Co-operation and Development (OECD) Test Guideline 318. UV–Vis peak positions and line shapes were consistent with published behavior for citrate-stabilized AgNPs of comparable size [37].

### 2.8. AgNP–Antibiotic Combination Testing: Checkerboard Assay

AgNP–antibiotic interactions were evaluated using a two-dimensional checkerboard microdilution assay in CAMHB on low-binding 96-well plates (final well volume 100 µL). Along the horizontal axis, AgNPs (reported as µg/mL total Ag) were dispensed in a twofold series, typically 0.5–128 µg/mL. Along the vertical axis, the partner antibiotic—oxacillin (primary) or ciprofloxacin (secondary)—was dispensed in a twofold series spanning its working range (see Section 2.5).

Each well received 50 µL of the 2× agent mixture and 50 µL of inoculum adjusted to 5 × 10^5^ CFU/mL. Plates were incubated at 35 ± 2 °C for 18–24 h and read visually to avoid optical interference from AgNPs. For every isolate, the MIC of each agent alone (single-agent rows/columns) and in combination (matrix wells) was recorded. To preserve dispersion stability and accuracy, plates were protected from light and edge wells were avoided to limit evaporation and nanoparticle ring effects.

Controls included sterility and growth controls, vehicle/dispersant controls, AgNO_3_ (ionic silver) at total-silver levels matched to AgNPs, and single-agent controls at the highest in-plate concentrations. All experiments were performed in biological duplicates with technical triplicates per condition.

Interaction was expressed as the FICI, calculated as:

(1) $$\mathrm{FICI}=\frac{{\mathrm{MIC}}_{\mathrm{AgNP}\;\mathrm{in}\;\mathrm{combination}}}{{\mathrm{MIC}}_{\mathrm{AgNP}\;\mathrm{alone}}}\times \frac{{\mathrm{MIC}}_{\mathrm{antibiotic}\;\mathrm{in}\;\mathrm{combination}}}{{\mathrm{MIC}}_{\mathrm{antibiotic}\;\mathrm{alone}}}$$

By convention, an FICI ≤ 0.5 indicates synergy, >0.5–1.0 additivity, >1.0–4.0 indifference, and >4.0 antagonism. When an MIC was off-scale (above the highest concentration tested), the top concentration was used to provide a conservative FICI estimate. FICI values were summarized as the median across technical replicates and the mean across biological duplicates. Representative combinations categorized as synergistic in the checkerboard were subsequently validated by time–kill assays (Section 2.9).

### 2.9. Time–Kill Kinetics

Time–kill assays were performed on a subset of representative MRSA isolates. Cultures (starting ~10^6^ CFU/mL) were exposed to AgNPs and to antibiotics—alone and in combination—at 0.5 × −1 × the MIC for each agent. Aliquots were collected at 0, 2, 4, 8, and 24 h, serially diluted, and plated for enumeration (CFU/mL). Synergy was defined as a ≥2 log_10_ CFU/mL reduction with the combination compared with the most active single agent at 24 h. Bactericidal activity was defined as a ≥3 log_10_ CFU/mL reduction from baseline.

### 2.10. Biofilm Assays (Subset Analysis)

For a subset of MRSA isolates (≥10), biofilm formation was quantified in flat-bottom 96-well plates under static conditions (24 h, 37 °C). Following treatment with AgNPs with or without antibiotics, biofilms were assessed by crystal violet staining (OD_590_) and by viable counts from biofilm resuspensions to estimate the minimum biofilm eradication concentration (MBEC). All conditions were tested in triplicate.

### 2.11. Resistance Evolution Experiment (Serial Passage)

To examine the potential for resistance development, selected MRSA isolates (≥10, spanning food categories and outlet types) were serially passaged once daily for 20–30 passages in CAMHB containing 0.5 × MIC AgNPs. Parallel arms included AgNPs plus sub-MIC oxacillin and oxacillin alone. Every five passages, MICs for AgNPs and the partner antibiotics were re-determined by broth microdilution. Frozen archives (−80 °C) were prepared at each interval. When AgNP MICs increased, we additionally measured growth rates, collected UV–Vis spectra from the spent medium to detect AgNP aggregation or surface-plasmon resonance (SPR) shifts, and assessed zeta potential. Selected ancestor–evolved pairs were retained for targeted sequencing of candidate loci. Ancestral and endpoint stocks (with intermediates, where indicated) were archived at −80 °C in glycerol for planned whole-genome sequencing.

### 2.12. Data Management and Statistical Analysis

Prevalence estimates were summarized as % (95% CI) by food category and outlet type; comparisons used χ^2^ or Fisher’s exact tests, as appropriate. MIC data were reported as MIC_50_/MIC_90_ and geometric means. Checkerboard results were summarized as median (IQR) FICI and categorized as synergy/additive/indifferent/antagonism using standard thresholds. Time–kill data were analyzed from log_10_ CFU/mL trajectories, with synergy and bactericidal activity adjudicated at 24 h as defined above.

Trends in resistance evolution were modeled using linear mixed-effects regression of log_2_(MIC) versus passage, with isolate as a random intercept; the Benjamini–Hochberg procedure controlled the false discovery rate. With n = 149, two-sided exact binomial 95% confidence intervals yield approximate half-widths of ±3.5, ±4.8, ±6.4, and ±7.4 percentage points at true prevalences of 5%, 10%, 20%, and 30%, respectively, indicating ~3–8 percentage-point precision across the typical range. Analyses were performed in R (v4.3+) and GraphPad Prism (v10) with α = 0.05.

### 2.13. Biosafety and Quality Assurance

All work was conducted under Biosafety Level 2 conditions with appropriate chemical safety for silver handling and waste disposal. ATCC controls were included in each run, and new media/consumable lots were verified against controls before study use. Instruments underwent routine calibration and internal quality control; any deviations triggered repeat testing or exclusion according to SOPs.

## 3. Results

### 3.1. Sample Characteristics and Recovery of S. aureus

We analyzed 149 RTE items collected between December 2024 and April 2025 using the cross-stratified sampling frame described in Table 1. After enrichment and selective/differential plating, presumptive *S. aureus* was recovered from 36 of the 149 samples (24.2%). Recovery differed by food category: meat-based 11/40 (27.5%), poultry-based 9/32 (28.1%), salads/vegetables 6/28 (21.4%), dairy-based 7/25 (28.0%), and bakery/sweets 3/24 (12.5%). Recovery also varied by outlet type: restaurants/takeaways 13/60 (21.7%), supermarkets/delis 11/54 (20.4%), and street vendors/markets 12/35 (34.3%). Exact proportions with two-sided 95% confidence intervals are reported in Table 2. To illustrate precision, exact binomial 95% confidence intervals are shown alongside all proportions. For a total sample size of 149, half-widths are approximately 5% of points when prevalence is near 10%, with narrower or wider intervals at lower or higher prevalences, respectively.

### 3.2. Confirmation of S. aureus, MRSA Prevalence, and MALDI Peak Signatures

Of the 36 presumptive isolates, 34 (94.4%) were confirmed as *S. aureus* by MALDI-TOF MS at species-level confidence (31 by direct smear; 3 after ethanol–formic acid extraction). Two isolates were reclassified as non-*aureus* staphylococci. Cefoxitin screening flagged 11 putative MRSA. Multiplex PCR detected *mecA* in 10 isolates and *mecC* in 0, yielding 10 MRSA and 24 MSSA overall. One cefoxitin-non-susceptible isolate was negative for *mecA*/*mecC* and PBP2a on repeat testing and was classified as MSSA. The corresponding MRSA prevalence among RTE items was 10/149 (6.7%; 95% CI, 3.3–11.8%). By food category, MRSA was detected in meat-based 4/40 (10.0%), poultry-based 3/32 (9.4%), salads/vegetables 1/28 (3.6%), dairy-based 1/25 (4.0%), and bakery/sweets 1/24 (4.2%). By outlet type, prevalence was 4/60 (6.7%) in restaurants/takeaways, 3/54 (5.6%) in supermarkets/delis, and 3/35 (8.6%) among street vendors/markets (Table 3).

**Figure 1:**
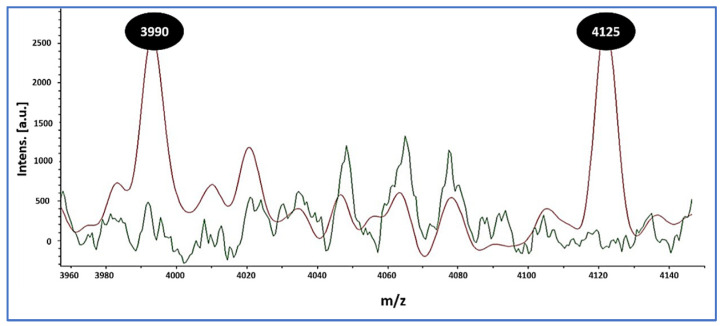
MALDI-TOF MS overlay distinguishing MRSA from MSSA. MRSA spectra (red) show reproducible peaks at mass-to-charge ratio (*m*/*z*) 3990 and 4125, whereas these peaks are absent in MSSA spectra (green). Inset: per-isolate spectra/spot counts (median [IQR]) and the proportion of MRSA exhibiting both peaks across ≥2 independent spots (10/10, 100%), with 0/24 (0%) MSSA showing either peak. These low-mass signatures differentiate MRSA from MSSA in this dataset and were used for spectral triage; definitive MRSA calls relied on a cefoxitin screen with *mecA*/*mecC* PCR.

In parallel, MALDI overlays revealed two reproducible low-mass peaks at *m*/*z* 3990 and 4125 that were present in all MRSA isolates (10/10, 100%) and absent in all MSSA isolates (0/24, 0%) (Figure 1). These signals were used strictly for preliminary triage. Final MRSA designations relied on cefoxitin-based screening with *mecA*/*mecC* confirmation, as detailed in the Methods.

### 3.3. Antimicrobial Susceptibility Profiles and Agreement Metrics (Vitek 2 AST-GP71)

Using the Vitek 2 Compact AST-GP71 panel, 34 *Staphylococcus aureus* isolates were profiled (MRSA, n = 10; MSSA, n = 24). Unless noted, interpretive categories and agreement metrics are referenced to BMD for oxacillin/cefoxitin, ciprofloxacin, and vancomycin (Table 4). As can be seen from Table 4, the β-lactam split was stark: oxacillin and benzylpenicillin (penicillin G) were 0/10 (0.0%) susceptible in MRSA, whereas oxacillin was 24/24 (100%) susceptible in MSSA and benzylpenicillin showed 2/24 (8.3%) susceptible and 22/24 (91.7%) resistant. Among fluoroquinolones, ciprofloxacin susceptibility was 1/10 (10.0%) in MRSA versus 22/24 (91.7%) in MSSA. For macrolides, erythromycin was 3/10 (30.0%) susceptible in MRSA and 19/24 (79.2%) in MSSA. Clindamycin (lincosamide) remained broadly active—7/10 (70.0%) MRSA and 22/24 (91.7%) MSSA susceptible. Gentamicin activity was high in both groups—9/10 (90.0%) MRSA and 23/24 (95.8%) MSSA susceptible. Rifampin susceptibility was 8/10 (80.0%) in MRSA and 22/24 (91.7%) in MSSA.

Several non-β-lactam agents retained uniform or near-uniform in vitro activity across the collection: linezolid, daptomycin, tigecycline, nitrofurantoin, quinupristin–dalfopristin, and vancomycin were 34/34 (100%) susceptible. Trimethoprim–sulfamethoxazole (TMP–SMX) was 33/34 (97.1%) susceptible overall, driven by a single resistant MSSA isolate (1/24, 4.2%). Agreement metrics were excellent. Categorical agreement (CA) reached 100.0% for most drugs (ciprofloxacin, daptomycin, linezolid, nitrofurantoin, oxacillin, quinupristin–dalfopristin, rifampin, tigecycline, TMP–SMX, vancomycin). CA was 97.1% for clindamycin and gentamicin (each with one minor error [mE] = 2.9%) and 97.1% for the cefoxitin screen (one major error [ME] = 4.2% among reference-susceptible MSSA). Benzylpenicillin showed 94.1% CA with two minor errors (5.9%). Very major errors (VME) were 0% for all agents. Essential agreement (EA) was not assessed for the cefoxitin screen and was not calculated for the remaining agents in this dataset.

These results confirm a clear MRSA–MSSA divide for β-lactams and ciprofloxacin, consistently high activity of multiple non-β-lactam agents against food-derived isolates, and robust categorical performance of the Vitek 2 AST-GP71 panel, with no VMEs and only isolated MEs/mEs.

### 3.4. In Vitro Activity of AgNPs: MICs and MBCs

Citrate-stabilized AgNPs demonstrated consistent inhibitory activity against all MRSA isolates (n = 10). MICs clustered within a narrow range of 8–32 µg/mL, with an MIC_50_ of 16 µg/mL and an MIC_90_ of 32 µg/mL (Table 5). Corresponding MBCs ranged from 16 to 128 µg/mL (median 32 µg/mL). In nine of ten isolates, the MBC/MIC ratio was ≤4, indicating bactericidal action at concentrations close to the MIC. One isolate required an MBC eightfold higher than its MIC (MBC/MIC = 8), consistent with a non-bactericidal effect under the test conditions.

Replicate determinations were highly reproducible, agreeing within ±1 twofold dilution, and all assay controls performed within acceptable limits. No clear association between MIC distribution and food category or outlet type was observed, likely due to the modest sample size. In summary, across the MRSA collection (n = 10), AgNPs displayed MICs ranging from 8 to 32 µg/mL (MIC_50_ = 16 µg/mL; MIC_90_ = 32 µg/mL). Corresponding MBCs spanned 16–128 µg/mL, yielding a median MBC/MIC ratio of two. Using the conventional bactericidal criterion (MBC/MIC ≤ 4), nine of ten isolates (90.0%) exhibited bactericidal activity. The MIC was defined as the lowest concentration preventing visible growth after 18–24 h, and the MBC as the lowest concentration achieving ≥99.9% reduction in viable counts. These results confirm that the tested AgNP formulation exerted strong and predominantly bactericidal effects against food-derived MRSA isolates.

### 3.5. AgNP–Antibiotic Interactions in MRSA (Checkerboard FICI)

**Figure 2:**
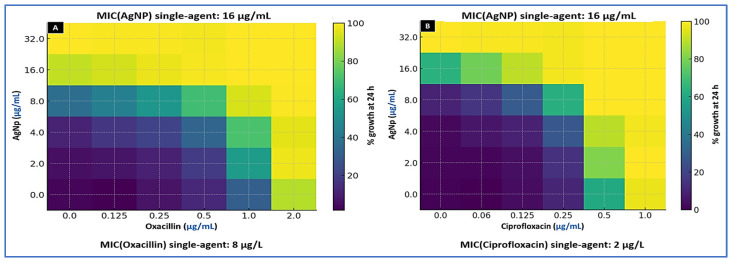
Checkerboard interaction heatmaps for citrate-stabilized silver nanoparticles (AgNPs) combined with (**A**) oxacillin and (**B**) ciprofloxacin against representative MRSA isolates. Axes show two-fold dilution series of AgNP (*y*-axis, µg/mL) and antibiotic (*x*-axis, µg/mL). Heatmap values represent percent growth at 24 h (visual read); the color bar uses numeric ticks (0, 25, 50, 75, 100%). Single-agent MICs for the isolate depicted are MIC (AgNP) = 16 µg/mL, MIC (oxacillin) = 8 µg/mL, and MIC (ciprofloxacin) = 2 µg/mL. FICI interpretation: synergy (≤0.5), additivity (>0.5–1.0), indifference (>1.0–4.0), and antagonism (>4.0). No antagonism was observed.

Checkerboard assays showed consistent potentiation of oxacillin by AgNPs against food-derived MRSA. Across ten isolates, the AgNP–oxacillin combination yielded synergy in 6/10 and additivity in 4/10, with no indifference or antagonism observed. The FICI had a median of 0.37 (IQR, 0.28–0.50), accompanied by 4–16-fold reductions in oxacillin MIC and 2–8-fold reductions in AgNP MIC compared with single-agent exposures. For AgNP–ciprofloxacin, synergy occurred in 3/10 isolates, additivity in 5/10, and indifference in 2/10 (no antagonism). The median FICI was 0.63 (IQR, 0.50–0.88), indicating an overall additive-to-synergistic effect. All interaction calls were reproducible across biological duplicates, with ≤1 twofold dilution difference between runs. Taken together, these data indicate that AgNPs re-sensitize a substantial fraction of MRSA to a β-lactam backbone and augment fluoroquinolone activity without detectable antagonism under the conditions tested (Figure 2; Supplementary Table S1).

### 3.6. Time–Kill Kinetics for AgNP Combinations

Time–kill assays were conducted on four MRSA isolates—two that showed synergy and two that showed additivity in checkerboard testing—to validate the dynamic effects of AgNP–antibiotic combinations using fixed concentrations rather than MIC multiples. For isolates with AgNP MICs of 8, 16, and 32 µg/mL, AgNPs were tested at 4 and 8 µg/mL, 8 and 16 µg/mL, and 16 and 32 µg/mL, respectively. Oxacillin was tested at 4 and 8 µg/mL, while ciprofloxacin was tested at 0.25 and 0.5 µg/mL (MIC 0.5 µg/mL), 0.5 and 1 µg/mL (MIC 1 µg/mL), or 1 and 2 µg/mL (MIC 2 µg/mL), matched to each isolate’s baseline susceptibility profile.

When AgNPs were combined with oxacillin, the regimen produced rapid and sustained bactericidal activity. At the higher combination dose (AgNP 8–32 µg/mL plus oxacillin 8 µg/mL), two isolates dropped below the limit of detection (10 CFU/mL) by 24 h, and the remaining two achieved ≥3-log_10_ CFU/mL reductions without regrowth. At the lower combination dose (AgNP 4–16 µg/mL plus oxacillin 4 µg/mL), three isolates showed reductions of approximately 2.3–3.8 log_10_ CFU/mL (synergy), and one isolate showed a 1.8 log_10_ CFU/mL reduction (additivity). In contrast, oxacillin alone (8 µg/mL) resulted in bacterial stasis or early regrowth, while AgNPs alone (8–32 µg/mL) produced only modest decreases of 0.5–1.2 log_10_ CFU/mL after 24 h.

**Figure 3:**
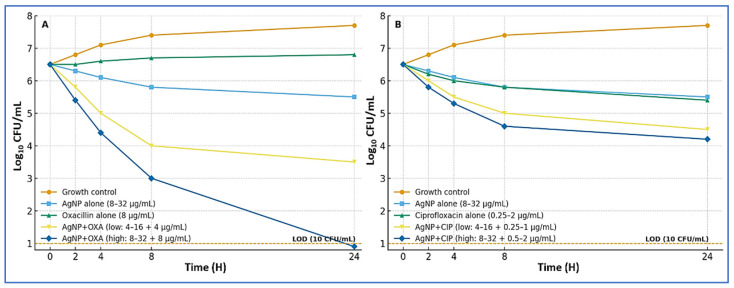
Time–kill kinetics of AgNP combinations against MRSA. (**A**) AgNPs with oxacillin. Curves show the growth control, AgNPs alone (8–32 µg/mL), oxacillin alone (8 µg/mL), and two combination regimens: low dose (AgNPs 4–16 µg/mL plus oxacillin 4 µg/mL) and high dose (AgNPs 8–32 µg/mL plus oxacillin 8 µg/mL). The high-dose combination produced rapid, sustained bactericidal activity, with some isolates reaching the limit of detection (LOD; 10 CFU/mL, dashed line) by 24 h. (**B**) AgNPs with ciprofloxacin. Curves show the growth control, AgNPs alone (8–32 µg/mL), ciprofloxacin alone (0.25–2 µg/mL), and two combination regimens: low dose (AgNPs 4–16 µg/mL plus ciprofloxacin 0.25–1 µg/mL) and high dose (AgNPs 8–32 µg/mL plus ciprofloxacin 0.5–2 µg/mL). Combinations showed additive-to-synergistic killing without evidence of antagonism, with an earlier onset of killing (4–8 h) and reduced regrowth compared with single agents.

When AgNPs were combined with ciprofloxacin (AgNP 4–32 µg/mL plus ciprofloxacin 0.25–2 µg/mL, dose-matched by isolate), the interaction was consistently additive-to-synergistic. Two isolates showed 1.5–2.7 log_10_ CFU/mL reductions, while two showed 1.0–1.4 log_10_ CFU/mL reductions, with no evidence of antagonism at any time point. Across all isolates, the combinations demonstrated an earlier onset of killing (within 4–8 h) and prevented regrowth more effectively than either agent alone. Representative kinetic curves are shown in Figure 3, while full isolate-level results—including 24 h Δlog_10_ CFU/mL changes, synergy/bactericidal adjudications, and limit-of-detection status—are detailed in Supplementary Table S2. All findings were reproducible across independent biological replicates, with pairwise differences ≤0.3 log_10_ CFU/mL at 24 h.

### 3.7. Experimental Evolution Under Sub-MIC AgNP Exposure

To model prolonged low-level pressure, isolates with baseline AgNP MICs of 8, 16, or 32 µg/mL were passaged once daily for 20 days in a CAMHB containing AgNPs at 4, 8, or 16 µg/mL, respectively. A parallel arm used the same AgNP levels plus oxacillin at 4 µg/mL. Both arms were followed by seven drug-free passages to assess stability of any changes. Under AgNP exposure alone, AgNP MICs increased modestly: the median change was twofold (IQR, 1–4-fold). Three isolates reached the pre-specified threshold for reduced susceptibility (≥4-fold rise), peaking at 4–8-fold above baseline. In two of these, the shift proved unstable and regressed to ≤2-fold after drug withdrawal; two isolates showed a durable increase and, together with their ancestors, were earmarked for targeted whole-genome sequencing in follow-up work.

**Figure 4:**
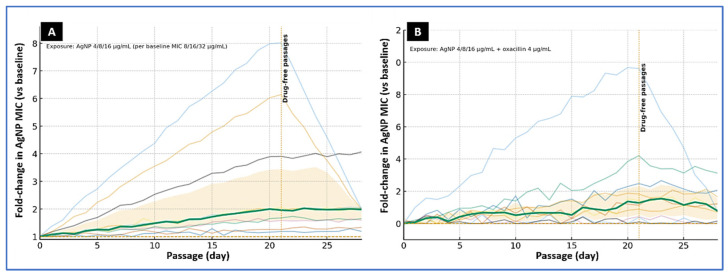
Experimental evolution of foodborne MRSA under sub-MIC AgNP exposure. (**A**) AgNP alone. Ten isolates were serially passaged for 20 days in cation-adjusted Mueller–Hinton broth (CAMHB) containing AgNPs at 4, 8, or 16 µg/mL, matched to baseline AgNP MICs of 8, 16, or 32 µg/mL, respectively. (**B**) AgNP plus β-lactam. Parallel passages used AgNPs at 4, 8, or 16 µg/mL together with oxacillin at 4 µg/mL. Curves display individual isolate trajectories (thin lines) as fold-change in AgNP MIC relative to baseline; the bold line indicates the median, with the shaded band showing the interquartile range (IQR). The vertical dotted line at day 21 marks the start of drug-free passages. Under AgNP exposure alone, several isolates showed multi-fold MIC increases with partial regression during drug-free culture, whereas the AgNP–oxacillin combination constrained MIC drift.

Adding oxacillin constrained adaptation. In the AgNP-plus-oxacillin arm (AgNP 4–16 µg/mL with oxacillin 4 µg/mL), the median MIC change was 0-fold (IQR, 0–2-fold), and the single twofold elevation returned to baseline during drug-free passage. Across conditions, MBCs tended to rise in step with MICs, but the MBC/MIC ratio remained ≤4 for most endpoint cultures, indicating preserved bactericidal behavior despite modest shifts in susceptibility. No consistent cross-changes were detected in oxacillin or ciprofloxacin MICs. Overall, sustained sub-MIC AgNP exposure selected small, often reversible increases in AgNP MIC, whereas pairing AgNPs with a β-lactam limited adaptation under the conditions tested (Figure 4).

## 4. Discussion

This study addresses a One Health priority by pairing real-world surveillance of RTE foods with rigorous confirmation of methicillin resistance and focused experiments on silver-based combination therapy. By integrating orthogonal identification methods—culture, MALDI-TOF MS, Vitek 2, and *mecA*/*mecC* PCR—with mechanistic assays (checkerboard synergy, time–kill kinetics, and experimental evolution), we move beyond prevalence reporting toward actionable intervention. We found that MRSA was present in 6.7% of RTE items, that citrate-stabilized AgNPs reliably restored or potentiated β-lactam activity with rapid, sustained bactericidal effects, and that prolonged sub-MIC exposure to silver produced only modest, often unstable MIC increases—further constrained when AgNPs were paired with oxacillin. These results support matrix-aware surveillance and combination-first strategies while cautioning against prolonged sub-therapeutic nanoparticle exposure.

From the 149 RTE items analyzed, *S. aureus* was recovered from 24.2% overall, with significantly higher recovery from animal-derived products and street vendors (≈1.7-fold higher than supermarkets/delis). This pattern mirrors global evidence implicating meat and dairy as high-risk matrices for staphylococcal contamination [5]. A recent meta-analysis estimated the global prevalence of MRSA in meats at ~3.7%, with the highest burden in the Eastern Mediterranean region [6]. Consistent findings have been reported in China, where Wang et al. [38] identified multidrug-resistant MRSA in retail foods and Wu et al. [3] documented notable contamination across diverse categories. In Europe, Basanisi et al. [39] reported 2.4% MRSA prevalence in southern Italian retail meats, while Normanno et al. [40] and Huber et al. [41] confirmed MRSA in dairy, meat, and RTE foods at variable rates. Beyond Europe, Weese et al. [42] detected MRSA in North American retail meats and linked isolates to both human- and livestock-associated clonal complexes, underscoring the zoonotic interface.

Regional studies support our outlet gradient: lapses in hygiene and infrastructure amplify contamination risk [43,44,45,46]. In Nigeria, Beshiru et al. [45] confirmed MRSA in RTE foods, raising consumer safety concerns, and in South Africa, Asiegbu et al. [47] found *S. aureus* in 31.8% of street-vended foods, highlighting informal markets as vulnerable nodes. In contrast, robust surveillance and cold-chain systems are associated with far lower rates. In Singapore, Aung et al. [48] detected MRSA in 2.2% of retail food isolates, and Zwe et al. [49] reported similarly low occurrence. Japan shows comparable patterns, with Kitai et al. [50] and Sato et al. [51] reporting consistently low MRSA prevalence in retail meats, attributed to strict hygiene and monitoring. These comparisons illustrate how infrastructural disparities shape exposure risk: informal or resource-limited markets remain persistent hotspots, whereas strong compliance frameworks can suppress MRSA in foods.

Biological plausibility for the category and outlet patterns is clear. Animal-derived foods often carry higher initial bioburden and undergo intensive handling, facilitating *S. aureus* survival and growth. By contrast, bakery items with low water activity and high sugar content naturally restrict *S. aureus* persistence. Operational factors—time to sale, temperature control, and surface sanitation—already shown to be inconsistent in Saudi food-service settings [43]—likely contribute to elevated contamination in street markets. These observations argue for matrix- and outlet-specific surveillance that prioritizes high-risk products and environments, with benchmarking against low-prevalence regions to demonstrate achievable suppression [49].

Orthogonal confirmation strengthens confidence in our workflow and highlights RTE foods as reservoirs of resistant staphylococci. The 6.7% MRSA prevalence observed here aligns with international reports: Somda et al. [52] found *S. aureus* in 70.8% of RTE foods in Burkina Faso with 14.3% MRSA; Islam et al. [53] identified MRSA in ~23% of Bangladeshi isolates; and Saber et al. [54] confirmed *mecA*-positive multidrug-resistant *S. aureus* in Egyptian RTE meats. MALDI-TOF MS provided rapid, accurate species confirmation and revealed two reproducible low-mass peaks (*m*/*z* 3990 and 4125) associated with MRSA in our dataset. Similar discriminatory features have been reported [55,56], and machine learning classifiers have achieved >87% MRSA–MSSA discrimination, though performance may vary by SCCmec type [57]. Practically, MALDI-based signatures are best used to triage isolates for targeted molecular confirmation, while *mecA*/*mecC* PCR remains indispensable.

AST profiles showed the classic β-lactam divide: MRSA remained non-susceptible to oxacillin/cefoxitin, whereas MSSA was oxacillin-susceptible but largely penicillin-resistant—consistent with MENA and global food-chain data [58]. To avoid clindamycin failure, routine screening for inducible macrolide–lincosamide–streptogramin B (MLSB) resistance by the CLSI-recommended D-test remains essential, given known discordance between erythromycin and clindamycin phenotypes [59]. Non-β-lactam trends also matched global reports: MRSA frequently resisted ciprofloxacin and macrolides, while glycopeptides and oxazolidinones were preserved [60]. Regionally, surveillance from Saudi Arabia and the UAE shows similar pressures in animal-derived RTE items and vendor settings [61]. Our automated system (Vitek 2, AST-GP71) demonstrated high categorical agreement, in line with multicenter evaluations (~98% CA; very-major-error ≤ 0.5%) [16]. Even so, confirmatory D-testing and *mecA*/*mecC* PCR remain prudent for critical decisions [62]. Accordingly, coupling automated AST with targeted molecular confirmation enables scalable food-chain surveillance and stewardship that reserves last-line agents for confirmed need.

Against confirmed food-derived MRSA, citrate-stabilized AgNPs showed consistent inhibitory potency (MIC 8–32 µg/mL; MIC_50_ 16; MIC_90_ 32) and were predominantly bactericidal (MBC/MIC ≤ 4 in 90%). Comparable ranges appear across diverse formulations: mycosynthesized particles (~27.7 nm) yielded MICs ~25 µg/mL [63]; green-synthesized *Curcuma longa* AgNPs produced low MICs [64]; and more resistant *S. aureus* required ~50/100 µg/mL MIC/MBC [65]. Because particle size, surface chemistry, and Ag^+^ release kinetics strongly determine potency, standardized reporting of size distributions, ζ-potential, and ion release is critical for cross-study comparison and risk assessment [66]. Our narrow MIC distribution suggests relatively consistent activity across strains, with a single isolate showing a higher MBC/MIC ratio of eight. This variability underscores the biological heterogeneity of *S. aureus* and the need to balance antimicrobial efficacy with safety and cost in translational applications [67].

Combination testing indicated that AgNPs can re-sensitize MRSA to a β-lactam scaffold. Checkerboards showed AgNP–oxacillin synergy in 60% and additivity in 40% of isolates (median FICI 0.37), with 4–16-fold reductions in oxacillin MIC and 2–8-fold reductions in AgNP MIC; AgNP–ciprofloxacin was additive-to-synergistic (median FICI 0.63) with no antagonism. Independent reports similarly describe AgNP–antibiotic potentiation against MRSA, e.g., nanosilver plus conventional agents yielding synergy and rapid killing [22], and enhanced effects in multimodality regimens (AgNPs + blue light + antibiotics) [24]. Mechanistically, silver’s multitarget action—inhibiting multiple essential proteins and pathways—provides a plausible basis for β-lactam re-sensitization and a higher barrier to stable resistance [21]. PK/PD-guided optimization of dosing ratios and schedules in food-relevant matrices is warranted to translate static synergy into robust operational interventions.

Time–kill assays confirmed that adding AgNPs converts oxacillin from static or failing monotherapy into a rapidly bactericidal regimen. At MIC-matched, fixed concentrations, AgNPs plus oxacillin achieved ≥3-log_10_ kills by 24 h in all four tested isolates (two reached the limit of detection), while monotherapies produced only stasis or ~0.5–1.2-log_10_ declines. AgNPs plus ciprofloxacin showed additive-to-synergistic trajectories with earlier onset of killing and suppressed regrowth, without antagonism—consistent with prior reports of accelerated kill and reduced rebound under combination exposure [22,24]. Together with the checkerboard data, these results support the practical value of combinations, contingent on dosing that maintains concentrations above synergy-enabling thresholds.

Experimental evolution under sub-MIC silver produced modest, often reversible, increases in AgNP MICs (median two-fold; IQR 1–4-fold), whereas co-exposure with oxacillin constrained drift and returned isolates to baseline after drug withdrawal. This pattern aligns with evidence that silver adaptation can emerge under specific exposure designs—sometimes via nanoparticle aggregation or altered envelope responses—but is not universally stable [26,68]. We detected no consistent collateral shifts in oxacillin or ciprofloxacin MICs; however, metal–antibiotic co-selection remains a credible risk in food and environmental settings. Longer-term surveillance should track ARG/HMRG linkages and fitness trade-offs [28,69].

Overall, our findings position RTE foods as credible MRSA exposure pathways—particularly animal-derived and street-vended items—and argue for matrix- and outlet-aware surveillance, strengthened hygiene/cold-chain controls, and scalable confirmation workflows (MALDI triage → automated AST → *mecA*/*mecC* PCR). AgNPs emerge as promising adjuvants that restore β-lactam efficacy and deliver rapid bactericidal activity, while combination use limits silver-specific MIC drift. Before field deployment, PK/PD optimization, standardized AgNP characterization, toxicity/residue assessments, and regulatory alignment are essential [21,66].

## 5. Limitations of the Study

This single-region, cross-sectional survey (n = 149) limits generalizability, seasonality assessment, and outlet-level inference; the MRSA subset (n = 10) constrains power for subgroup analyses. MALDI-TOF MRSA-associated peaks were identified in a small dataset without external or interlaboratory validation and should be treated as triage cues pending broader verification. AST relied primarily on Vitek 2 rather than comprehensive BMD across all agents, and SCCmec/clone-level correlates were not resolved. Whole-genome sequencing of evolved lines was not performed, limiting mechanistic insight into silver-response pathways and potential co-selection. Baseline and endpoint aliquots were preserved, and a follow-up is pre-specified to sequence the two isolates with the largest durable MIC increases alongside their ancestors. Formal fitness assays were beyond scope. Finally, safety, residue, and regulatory considerations (cytotoxicity, ecotoxicity, and compliance for food-contact materials) were not evaluated and require dedicated study.

## 6. Conclusions

Across 149 RTE foods from Al-Qassim, *S. aureus* occurred in 24.2% and MRSA in 6.7%, concentrated in animal-derived items and street-vendor outlets. An orthogonal workflow—culture, MALDI-TOF MS, Vitek 2, and *mecA*/*mecC* PCR—delivered rapid, reliable confirmation; reproducible low-mass MALDI peaks supported triage, while PCR provided definitive calls. Susceptibility testing showed the expected β-lactam split with preserved activity of last-line agents, supporting automated AST augmented by targeted D-testing and *mec* gene confirmation. Citrate-stabilized AgNPs were consistently potent (MIC 8–32 µg/mL; MIC_50_ 16; MIC_90_ 32) and predominantly bactericidal, and in combination produced frequent synergy with oxacillin and additive-to-synergistic effects with ciprofloxacin that translated into rapid, sustained killing without antagonism. Sub-MIC evolution selected only modest, often reversible increases in silver MIC that were curtailed by β-lactam co-exposure. These findings justify matrix- and outlet-aware surveillance, support MALDI-assisted triage integrated with automated AST and *mecA*/*mecC* PCR, and nominate standardized AgNP formulations as promising β-lactam adjuvants for food-adjacent risk reduction, pending in situ efficacy, safety, and residue evaluation. Future work will incorporate whole-genome sequencing of selected evolved pairs to clarify genetic drivers of silver susceptibility changes and interactions with β-lactam exposure.

## Figures and Tables

**Table 1:** Allocation of 149 RTE food samples across categories and outlet types, Al-Qassim. Sampling period: December 2024–April 2025.

Category→/Outlet	Restaurants	Supermarkets	Street Vendors	Total
Meat-based RTE	18	14	8	40
Poultry-based RTE	14	11	7	32
Salads/vegetables RTE	10	10	8	28
Dairy-based RTE	8	11	6	25
Bakery/sweets	10	8	6	24
Column total	60	54	35	149

**Table 2:** Recovery of presumptive *S. aureus* from RTE foods by category and outlet type.

**By Food Category**			
**Stratum**	**Positive/Total**	**Positive (%)**	**95% CI (%)**
Meat-based RTE	11/40	27.5	14.6–43.9
Poultry-based RTE	9/32	28.1	13.7–46.7
Salads/vegetables RTE	6/28	21.4	8.3–41.0
Dairy-based RTE	7/25	28.0	12.1–49.4
Bakery/sweets	3/24	12.5	2.7–32.4
**By Outlet Type**			
**Stratum**	**Positive/Total**	**Positive (%)**	**95% CI (%)**
Restaurants/takeaways	13/60	21.7	12.1–34.2
Supermarkets/delis	11/54	20.4	10.6–33.5
Street vendors/markets	12/35	34.3	19.1–52.2

Notes: Sampling period: December 2024–April 2025. Proportions are the percentage of samples yielding presumptive *S. aureus* after enrichment and selective/differential plating. Confidence intervals (CIs) are two-sided exact binomial (Clopper–Pearson) 95% CIs.

**Table 3:** Prevalence of MRSA among RTE food samples by category and outlet type.

**By Food Category**			
**Stratum**	**MRSA/Total**	**MRSA (%)**	**95% CI (%)**
Meat-based RTE	4/40	10.0	2.8–23.7
Poultry-based RTE	3/32	9.4	2.0–25.0
Salads/vegetables RTE	1/28	3.6	0.1–18.3
Dairy-based RTE	1/25	4.0	0.1–20.4
Bakery/sweets	1/24	4.2	0.1–21.1
**By Outlet Type**			
**Stratum**	**MRSA/Total**	**MRSA (%)**	**95% CI (%)**
Restaurants/takeaways	4/60	6.7	1.8–16.2
Supermarkets/delis	3/54	5.6	1.2–15.4
Street vendors/markets	3/35	8.6	1.8–23.1

Notes: MRSA defined as *mecA* or *mecC* detection with supportive phenotype. Two-sided exact (Clopper–Pearson) 95% confidence intervals. Percentages are of total samples within each stratum.

**Table 4:** Antimicrobial susceptibility of *S. aureus* from RTE foods (MRSA n = 10; MSSA n = 24) and agreement metrics for Vitek 2 AST-GP71 versus reference methods.

Antimicrobial Agent	MRSA				MSSA				Total				CA		VME		ME		mE	
	S		R		S		R		S		R									
	n	%	n	%	n	%	n	%	n	%	n	%	n	%	n	%	n	%	n	%
Cefoxitin (screen)	0	0.0	10	100.0	23	95.8	1	4.2	23	67.6	11	32.4	33	97.1	0/10	0.0	1	4.2	0	0.0
Ciprofloxacin	1	10.0	9	90.0	22	91.7	2	8.3	23	67.6	11	32.4	34	100.0	0	0.0	0	0.0	0	0.0
Clindamycin	7	70.0	3	30.0	22	91.7	2	8.3	29	85.3	5	14.7	33	97.1	0	0.0	0	0.0	1	2.94
Daptomycin	10	100.0	0	0.0	24	100.0	0	0.0	34	100.0	0	0.0	34	100.0	0	0.0	0	0.0	0	0.0
Erythromycin	3	30.0	7	70.0	19	79.2	5	20.8	22	64.7	12	35.3	34	100.0	0	0.0	0	0.0	0	0.0
Gentamicin	9	90.0	1	10.0	23	95.8	1	4.2	32	94.1	2	5.9	33	97.1	0	0.0	0	0.0	1	2.94
Linezolid	10	100.0	0	0.0	24	100.0	0	0.0	34	100.0	0	0.0	34	100.0	0	0.0	0	0.0	0	0.0
Nitrofurantoin	10	100.0	0	0.0	24	100.0	0	0.0	34	100.0	0	0.0	34	100.0	0	0.0	0	0.0	0	0.0
Oxacillin	0	0.0	10	100.0	24	100.0	0	0.0	24	70.6	10	29.4	34	100.0	0	0.0	0	0.0	0	0.0
Benzylpenicillin	0	0.0	10	100.0	2	8.3	22	91.7	2	5.9	32	94.1	32	94.12	0	0.0	0	0.0	2	5.88
Quinupristin–dalfopristin	10	100.0	0	0.0	24	100.0	0	0.0	34	100.0	0	0.0	34	100.0	0	0.0	0	0.0	0	0.0
Rifampin	8	80.0	2	20.0	22	91.7	2	8.3	30	88.2	4	11.8	34	100.0	0	0.0	0	0.0	0	0.0
Tigecycline	10	100.0	0	0.0	24	100.0	0	0.0	34	100.0	0	0.0	34	100.0	0	0.0	0	0.0	0	0.0
Trimethoprim–sulfamethoxazole	10	100.0	0	0.0	23	95.8	1	4.2	33	97.1	1	2.9	34	100.0	0	0.0	0	0.0	0	0.0
Vancomycin	10	100.0	0	0.0	24	100.0	0	0.0	34	100.0	0	0.0	34	100.0	0	0.0	0	0.0	0	0.0

**Table 5:** In vitro activity of AgNPs against MRSA: MIC, MBC, killing classification, fold-change vs. MIC_50_, and baseline oxacillin/ciprofloxacin MICs (broth microdilution).

Isolate ID	Food Category	Isolate Type	AgNP MIC (µg/mL)	Fold-Change vs. MIC_50_ (16 µg/mL)	AgNP MBC (µg/mL)	MBC/MIC	Killing Classification	Baseline MICs (µg/L): OXA; CIP
MRSA-01	Meat-based	Restaurant/Takeaways	16	1.0	32	2	Bactericidal	8; 2
MRSA-02	Meat-based	Supermarket/Deli	16	1.0	32	2	Bactericidal	8; 0.5
MRSA-03	Meat-based	Street vendor/Market	32	2.0	64	2	Bactericidal	8; 2
MRSA-04	Meat-based	Restaurant/Takeaways	8	0.5	16	2	Bactericidal	8; 1
MRSA-05	Poultry-based	Supermarket/Deli	16	1.0	32	2	Bactericidal	8; 2
MRSA-06	Poultry-based	Restaurant/Takeaways	8	0.5	16	2	Bactericidal	8; 2
MRSA-07	Poultry-based	Street vendor/Market	16	1.0	64	4	Bactericidal	8; 1
MRSA-08	Salads/vegetables	Street vendor/Market	32	2.0	64	2	Bactericidal	8; 2
MRSA-09	Dairy-based	Supermarket/Deli	16	1.0	32	2	Bactericidal	8; 2
MRSA-10	Bakery/sweets	Restaurant/Takeaways	16	1.0	128	8	Non-bactericidal (isolate)	8; 2

Reference method: broth microdilution (CLSI M07/M100) for oxacillin/cefoxitin, ciprofloxacin, and vancomycin; Vitek 2 results shown for comparison. AgNP batch: same lot characterized in Section 2.7 (dynamic light scattering hydrodynamic diameter, ζ-potential, and ICP-MS total Ag).

## Data Availability

The original contributions presented in this study are included in the article and Supplementary Material. Further inquiries can be directed to the corresponding author.

## Supplementary Materials

The following supporting information can be downloaded at: https://www.mdpi.com/article/10.3390/microorganisms13102393/s1, Table S1: Isolate-level checkerboard interactions of silver nanoparticles (AgNPs) with oxacillin and ciprofloxacin against MRSA; Table S2: Time–kill outcomes for silver nanoparticle (AgNP) combinations against MRSA (subset, n = 4).
