# Supplementary material for: Combating Foodborne MRSA: Identification and Silver Nanoparticle-Based Antibacterial Strategies with Antibiotic Synergy and Resistance Evolution Assessment
Source: Microorganisms. 2025 Oct 18;13(10):2393. doi: 10.3390/microorganisms13102393 (PMC12565767; doi:10.3390/microorganisms13102393)
Supplement: Supplementary file 1 [file microorganisms-13-02393-s001.zip › microorganisms-3915669-supplementary.pdf]

Table S1. Isolate-level checkerboard interactions of silver nanoparticles (AgNPs) with oxacillin and ciprofloxacin against MRSA.

Single-agent MICs were determined as described (AgNPs reported as total silver, µg/mL). Combination MICs reflect the lowest concentrations yielding no visible growth at 24 h. FICI = FIC (AgNP) + FIC (antibiotic), where FIC (agent) = (MIC in combination) ÷ (MIC alone). Interaction categories: synergy (FICI ≤ 0.5), additivity (>0.5–1.0), indifference (>1.0–4.0), antagonism (>4.0).

Isolate ID	AgNP MIC (µg/mL)	Oxacillin MIC (µg/mL) *	Ciprofloxacin MIC (µg/mL)	AgNP+Oxacillin: AgNP in combo (µg/mL)	AgNP+Oxacillin: Oxacillin in combo (µg/mL)	AgNP+Oxacillin: FICI	AgNP+Oxacillin: Interaction	Oxacillin MIC fold-reduction	AgNP+Ciprofloxacin: AgNP in combo (µg/mL)	AgNP+Ciprofloxacin: Ciprofloxacin in combo (µg/mL)	AgNP+Ciprofloxacin: FICI	AgNP+Ciprofloxacin: Interaction	Ciprofloxacin MIC fold-reduction
MRSA-01	16	8	2	4	1	0.38	Synergy	8-fold	4	0.25	0.38	Synergy	8-fold
MRSA-02	16	8	0.5	8	4	1.00	Additive	2-fold	8	0.25	1.00	Additive	2-fold
MRSA-03	32	8	2	8	1	0.38	Synergy	8-fold	16	1	1.00	Additive	2-fold
MRSA-04	8	8	1	2	1	0.38	Synergy	8-fold	2	0.25	0.50	Synergy	4-fold
MRSA-05	16	8	2	4	2	0.50	Synergy	4-fold	8	1	1.00	Additive	2-fold
MRSA-06	8	8	2	4	4	1.00	Additive	2-fold	2	0.5	0.50	Synergy	4-fold
MRSA-07	16	8	1	8	2	0.75	Additive	4-fold	16	1	2.00	Indifferent	1-fold
MRSA-08	32	8	2	8	1	0.38	Synergy	8-fold	16	1	1.00	Additive	2-fold
MRSA-09	16	8	2	4	1	0.38	Synergy	8-fold	16	2	2.00	Indifferent	1-fold
MRSA-10	16	8	2	8	2	0.75	Additive	4-fold	8	1	1.00	Additive	2-fold

* For isolates with oxacillin MICs at or above the test range, the highest in-plate concentration (8 µg/mL) was used as the working single-agent MIC for conservative FICI estimation. Fold-reduction = (single-agent MIC) ÷ (combination MIC) for the antibiotic component.

Table S2. Time–kill outcomes for silver nanoparticle (AgNP) combinations against MRSA (subset, n = 4).

Reductions are reported as Δlog10 CFU/mL at 24 h relative to baseline. Synergy was predefined as a ≥2-log$_{10}$ reduction versus the most active single agent; "Borderline" indicates ~2-log$_{10}$ advantage without meeting the threshold across replicates. Bactericidal activity was defined as a ≥3-log$_{10}$ reduction from baseline. LOD indicates counts below the limit of detection (10 CFU/mL).

Isolate	AgNP alone (µg/mL) Δlog10	Oxacillin alone 8 µg/mL Δlog10	Ciprofloxacin alone (µg/mL) Δlog10	AgNP+Oxacillin (LOW) AgNP + OXA (µg/mL) Δlog10	Synergy?	Bactericidal?	LOD?	AgNP+Oxacillin (HIGH) AgNP + OXA (µg/mL) Δlog10	Synergy?	Bactericidal?	LOD?	AgNP+Ciprofloxacin (LOW) AgNP + CIP (µg/mL) Δlog10	Synergy?	Bactericidal?	LOD?	AgNP+Ciprofloxacin (HIGH) AgNP + CIP (µg/mL) Δlog10	Synergy?	Bactericidal?	LOD?
MRSA-01	16 ǀ -1.1	-0.1	2 ǀ -1.0	8 + 4 ǀ -3.2	Yes	Yes	No	16 + 8 ǀ -3.7	Yes	Yes	Yes	8 + 1 ǀ -2.2	Borderline	No	No	16 + 2 ǀ -2.7	Yes	No	No
MRSA-02	16 ǀ -0.6	0.0	0.5 ǀ -0.9	8 + 4 ǀ -2.0	No	No	No	16 + 8 ǀ -3.1	Yes	Yes	No	8 + 0.25 ǀ -1.1	No	No	No	16 + 0.5 ǀ -1.3	No	No	No
MRSA-04	8 ǀ -0.9	0.1	1 ǀ -1.1	4 + 4 ǀ -2.8	Yes	No	No	8 + 8 ǀ -3.4	Yes	Yes	Yes	4 + 0.5 ǀ -1.8	No	No	No	8 + 1 ǀ -2.2	No	No	No
MRSA-10	16 ǀ -0.7	0.1	2 ǀ -0.8	8 + 4 ǀ -1.8	No	No	No	16 + 8 ǀ -3.2	Yes	Yes	No	8 + 1 ǀ -1.1	No	No	No	16 + 2 ǀ -1.3	No	No	No

LOW dose = AgNP at half the isolate's MIC and antibiotic at 4 µg/mL (oxacillin) or at half the isolate's MIC (ciprofloxacin). HIGH dose = AgNP at the isolate's MIC and antibiotic at 8 µg/mL (oxacillin) or at the isolate's MIC (ciprofloxacin).
